# Magnetic resonance imaging identifies early effects of sunitinib treatment in human melanoma xenografts

**DOI:** 10.1186/1756-9966-32-93

**Published:** 2013-11-19

**Authors:** Jon-Vidar Gaustad, Viktoria Pozdniakova, Tord Hompland, Trude G Simonsen, Einar K Rofstad

**Affiliations:** 1 Department of Radiation Biology, Group of Radiation Biology and Tumor Physiology, Institute for Cancer Research, Oslo University Hospital, Montebello, Oslo N-0310, Norway

**Keywords:** Sunitinib, Antiangiogenic treatment, Hypoxia, DW-MRI, DCE-MRI

## Abstract

**Background:**

Antiangiogenic treatment may change the tumor microenvironment and hence influence the effect of conventional therapies. The potential of diffusion weighted magnetic resonance imaging (DW-MRI) and dynamic contrast enhanced MRI (DCE-MRI) in assessing microenvironmental effects of sunitinib treatment was investigated in this preclinical study.

**Methods:**

Sunitinib-treated and untreated A-07 tumors were subjected to DW-MRI and DCE-MRI, and parametric images of ADC and *K*^trans^ were produced. Microvascular density, hypoxic fraction, and necrotic fraction were assessed from immunohistochemical preparations, and tumor interstitial fluid pressure (IFP) was assessed with probe measurement.

**Results:**

Sunitinib-treated tumors showed reduced microvascular density, increased hypoxic fraction, increased necrotic fraction, increased ADC, and reduced *K*^trans^, but did not differ from untreated tumors in growth rate and IFP.

**Conclusions:**

Sunitinib treatment affected the tumor microenvironment without affecting tumor size. DW-MRI and DCE-MRI were sensitive to the sunitinib-induced changes in the tumor microenvironment.

## Background

Several antiangiogenic drugs are being investigated, including endogenous inhibitors of angiogenesis [1], monoclonal antibodies against pro-angiogenic factors or their receptors [2,3], and small molecule tyrosine kinase inhibitors which may target multiple pro-angiogenic receptors [4]. The antiangiogenic agents are generally not cytotoxic, and treatment-induced reductions in tumor size often appear late compared to vascular effects [5]. It is therefore recognized that functional parameters are more appropriate than tumor size for evaluating early effects of antiangiogenic treatment [6].

Antiangiogenic therapy may inhibit tumor growth significantly when used as a single treatment modality, but the therapeutic benefit may be even greater when used in combination with conventional treatment modalities such as radiation and chemotherapy [7]. Tumor response to radiation and chemotherapy can be significantly affected by the tumor microenvironment. Tumors with extensive hypoxia are more resistant to radiation and some forms of chemotherapy, and elevated interstitial fluid pressure (IFP) may reduce the uptake of chemotherapeutic drugs [8,9]. Antiangiogenic treatment has been reported to improve oxygenation and reduce IFP in some tumor models [2,3] and to induce hypoxia in others [10,11]. The reasons for these different effects are not clear, but the effects have important implications for combination therapies. Careful monitoring of the tumor microenvironment during antiangiogenic treatment may help to optimize the timing of combination therapies.

Tumor response to antiangiogenic treatment has been evaluated with diffusion weighted magnetic resonance imaging (DW-MRI) and dynamic contrast-enhanced MRI (DCE-MRI) [6,12]. DW-MRI is sensitive to the Brownian motion of water molecules which is restricted by cell membranes and extracellular fibers in tissues [12]. The apparent diffusion coefficient (ADC) is often used to quantify DW-MRI data, and this parameter has been shown to reflect cell density and to be sensitive to necrotic tissue in untreated tumors [12,13]. Moreover, both reductions and increases in tumor ADC have been reported after antiangiogenic treatment [14,15]. In DCE-MRI, the uptake of a paramagnetic contrast agent is studied by imaging tumors before and multiple times within a few minutes after the injection of the contrast agent. The transfer rate constant, *K*^trans^, can be estimated by using the generalized pharmacokinetic model of Tofts to analyze DCE-MRI series [16]. *K*^trans^ generally reflects blood perfusion and the vessel permeability - vessel surface area product [17]. When using low molecular weight contrast agents like Gd-DTPA (550 Da), *K*^trans^ has been shown to reflect blood perfusion in untreated tumors with high vessel permeability [18]. Reductions in *K*^trans^ or *K*^trans^ -related parameters have been reported in most studies evaluating tumor response to antiangiogenic agents with DCE-MRI [6]. A weakness in many of the studies evaluating tumor response to antiangiogenic treatment with DW-MRI and/or DCE-MRI is that treatment-induced effects on the tumor microenvironment were not assessed with non-MR techniques. Consequently, it is not always clear how the changes in MR-derived parameters were related to the tumor microenvironment.

Sunitinib is a small molecule tyrosine kinase inhibitor which targets vascular endothelial growth factor receptors 1-3 (VEGFR-1, -2, and -3), platelet-derived growth factor receptors α-β (PDGFR-α and PDGFR-β), stem cell growth factor receptor (c-KIT), and fms-like tyrosine kinase receptor 3 (FLT 3) [19]. Sunitinib has been shown to prolong progression-free and overall survival in patients with imatinib-refractory gastrointestinal stromal tumor, metastatic renal cell carcinoma, and progressive, well-differentiated pancreatic neuroendocrine tumor in clinical phase III trials, and has been approved by the US Food and Drug Administration for these indications [20-22]. In the current study we evaluated the effect of sunitinib treatment on the tumor microenvironment by using histological techniques to assess microvessels, tumor hypoxia, and tumor necrosis and probe measurement to assess tumor IFP. We also evaluated the effect of sunitinib treatment with DW-MRI and DCE-MRI. We report that sunitinib treatment increased ADC and reduced *K*^trans^, reflecting sunitinib-induced tumor necrosis and sunitinib-induced reductions in tumor microvascular density and oxygenation.

## Methods

### Mice and tumors

Adult (8-12 weeks of age) female BALB/c*-nu/nu* mice, bred at our research institute, were used as host animals for xenografted tumors. Animal care and experimental procedures were approved by the Institutional Committee on Research Animal Care and were performed in accordance with the Interdisciplinary Principles and Guidelines for the Use of Animals in Research, Marketing, and Education (New York Academy of Sciences, New York, NY, USA). The experiments were performed with tumors of the amelanotic human melanoma A-07, established and characterized as described previously [23]. A-07 cells were obtained from our frozen stock and were cultured in RPMI-1640 medium (25 mM HEPES and L-glutamine) supplemented with 13% bovine calf serum, 250 mg/l penicillin, and 50 mg/l streptomycin. Approximately 3.5 × 10^5^ cells in 10 μl of Hanks’ balanced salt solution (HBSS) were inoculated intradermally in the hind leg by using a 100-μl Hamilton syringe. Tumor volume (*V*) was calculated as *V* = (*π/*6) *× a × b*^2^, where *a* is the longer and *b* is the shorter of two perpendicular diameters, measured with calipers.

### Sunitinib treatment

Sunitinb L-malate (LC Laboratories, Woburn, MA, USA) was dissolved in hydrochloric acid (1.0 molar ratio of sunitinib). Polysorbate 80 (0.5%; Sigma-Aldrich, Schnelldorf, Germany), polyethylene Glycol 300 (10%; Sigma-Aldrich), sodium hydroxide (to adjust pH to 3.5), and sterile water were added to the solution. Mice were treated with 40 mg/kg/day sunitinib or vehicle for 4 days, by oral administration.

### Anesthesia

MRI and IFP measurements were carried out with anesthetized mice. Fentanyl citrate (Janssen Pharmaceutica, Beerse, Belgium), fluanisone (Janssen Pharmaceutica), and midazolam (Hoffmann-La Roche, Basel, Switzerland) were administered intraperitoneally in doses of 0.63 mg/kg, 20 mg/kg, and 10 mg/kg, respectively. The body core temperature of the mice was kept at 37-38°C during MRI and IFP measurements by using a thermostatically regulated heating pad.

### MRI

MRI was performed by using a 1.5-T whole-body clinical scanner (Signa; General Electric, Milwaukee, WI, USA) and a slotted tube resonator transceiver coil constructed for mice. The tumors were positioned in the isocenter of the magnet and were imaged axially in a single section through the tumor center.

DW-MRI was carried out by applying a diffusion-weighted single-shot fast spin echo sequence with ETL = 84 and TR = 5002 ms. The diffusion weighted images were recorded at a spatial resolution of 0.39 × 0.39 × 2.0 mm^3^ by using an image matrix of 256 × 256, a field of view of 10 × 10 cm^2^, and 5-10 excitations. Diffusion sensitization gradients were applied in six non-collinear directions with the following x, y, and z physical gradient combinations: [1 0 1], [-1 0 1], [0 1 1], [0 1-1], [1 1 0], [-1 1 0]. Three different diffusion-weightings with diffusion encoding constants of *b* = 200, 400, and 800 s/mm^2^ and corresponding echo times of TE = 85, 95.5, and 108.9 ms were used. An image without diffusion weighting (*b* = 0) was recorded for each TE value to compensate for the different TEs associated with the different *b* values. The total scan time of our DW-MRI method was ~ 10 min. ADC maps were produced with in-house-made software developed in Matlab. Briefly, the directional diffusion images were averaged on a voxel-by-voxel basis to non-directional diffusion images. ADC values were calculated for each voxel by fitting signal intensities (*S*) to the mono-exponential model equation:

$$\log\left(\frac{S\left(b,\mathrm{TE}\right)}{S\left(b=0,\mathrm{TE}\right)}\right)=-b\cdot \mathrm{ADC}+c$$

by using a linear least square fit algorithm. The signal decay of a large number of voxels was investigated to verify that the mono-exponential model gave good fits to the data. The fits generally had a correlation coefficient of 0.98 - 0.99.

DCE-MRI was carried out as described earlier [24]. Briefly, Gd-DTPA (Schering, Berlin, Germany), diluted to a final concentration of 0.06 M, was administered in the tail vein of mice in a bolus dose of 5.0 ml/kg during a period of 5 s. Two calibration tubes, one with 0.5 mM Gd-DTPA in 0.9% saline and the other with 0.9% saline only, were placed adjacent to the mice in the coil. The tumors and the calibration tubes were imaged at a spatial resolution of 0.23 × 0.23 × 2.0 mm^3^ by using an image matrix of 256 × 128, a field of view of 6 × 3 cm^2^, and one excitation. Two types of spoiled gradient recalled images were recorded: proton density images (TR = 900 ms, TE = 3.2 ms, and α_PD_ = 20) and *T*_*1*_-weighted images (TR = 200 ms, TE = 3.2 ms, and α_T1_ = 80). The durations of the imaging sequences were 64 and 14 s, respectively. Two proton density images and three *T*_*1*_-weighted images were acquired before Gd-DTPA was administered. After the administration of Gd-DTPA, *T*_*1*_-weighted images were recorded every 14 s for 15 min. Gd-DTPA concentrations were calculated from signal intensities by using the method of Hittmair et al. [25]. The DCE-MRI series were analyzed on a voxel-by-voxel basis by using the arterial input function of Benjaminsen et al. [24] and the Tofts pharmacokinetic model [16] to produce parametric images of *K*^trans^.

### IFP measurements

IFP was measured by using a Millar SPC 320 catheter equipped with a 2F Micro-Tip transducer with diameter 0.66 mm (Millar Instruments, Houston, TX) [26]. The catheter was connected to a computer via a Millar TC-510 control unit and a model 13-66150-50 preamplifier (Gould Instruments, Cleveland, OH). IFP was measured in the center of the tumors by placing the catheter 5-10 mm from the tumor surface. Marks on the catheter assured correct positioning of the sensor, and a single measurement was carried out in each tumor. Only IFP measurements with stable readings for 3-5 minutes were accepted, and the measurements lasted for 10-20 minutes. Data acquisition was carried out by using LabVIEW software (National Instruments, Austin, TX).

### Hypoxia, necrosis, and microvessels

CD31 was used as a marker for endothelial cells and pimonidazole [1-[(2-hydroxy-3-piperidinyl)-propyl]-2-nitroimidazole] was used as a hypoxia marker. Pimonidazole was dissolved in 0.9% sodium chloride and administered intraperitoneally at a dose of 30 mg/kg. The tumors were resected and fixed in phosphate-buffered 4% paraformaldehyde approximately 4 hours after the pimonidazole administration. Immunohistochemistry was done by using a peroxidase-based indirect staining method [27]. An anti-pimonidazole rabbit polyclonal antibody (gift from Prof. J.A. Raleigh, Department of Radiation Oncology, University of North Carolina School of Medicine, Chapel Hill, NC) or an anti-CD31 rabbit polyclonal antibody (Abcam, Cambridge, United Kingdom) was used as primary antibody. Diaminobenzidine was used as chromogen, and hematoxylin was used for counterstaining. Hypoxic fraction was defined as the area fraction showing positive pimonidazole staining (hypoxic fraction = pimonidazole positive area/viable tissue area·100%) and necrotic fraction was defined as the area fraction showing necrotic tissue (necrotic fraction = necrotic tissue area/total area·100%). The area fraction showing positive pimonidazole staining and the area fraction showing necrotic tissue were determined by image analysis. Microvascular density was defined as the number of microvessel profiles per mm^2^ of viable tumor tissue (microvascular density = number of microvessel profiles/viable tissue area). The number of microvessel profiles was scored manually in immunohisochemical preparations stained with anti-CD31 antibody.

### Statistical analysis

Statistical comparisons of data were carried out by the Student’s *t* test when the data complied with the conditions of normality and equal variance. Under other conditions, comparisons were done by nonparametric analysis using the Mann-Whitney rank sum test. Probability values of *P* < 0.05, determined from two-sided tests, were considered significant. The statistical analysis was performed by using the SigmaStat statistical software (SPSS Science, Chicago, IL, USA).

## Results

**Figure 1:**
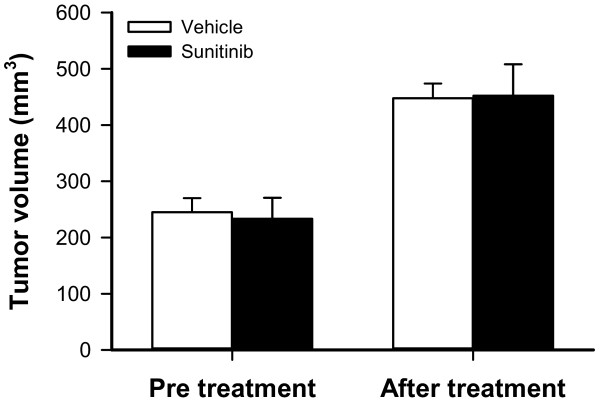
**Sunitinib treatment did not affect tumor growth.** Tumor size before and after 4 days of treatment in mice given vehicle (white colomns) or sunitinib (black columns). Columns, means of 14-15 A-07 tumors, bars SEM.

A-07 tumors were divided into groups with matched tumor sizes to receive sunitinib treatment or no treatment (vehicle). Tumors in both groups grew during the 4-day treatment period (Figure 1). After the treatment, sunitinib-treated tumors did not differ from untreated tumors in size (Figure 1; *P* > 0.05), indicating that this short-term treatment did not affect tumor growth.

**Figure 2:**
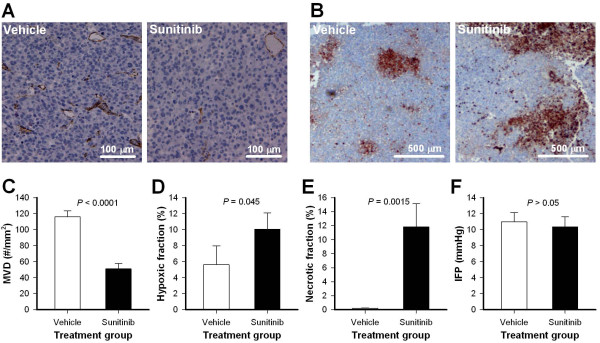
**Sunitinib treatment affected tumor physiology. A-B**, representative immunohistochemical preparations stained with anti-CD31 antibody to visualize microvessels **(A)** or anti-pimonidazole antibody to visualize hypoxic regions **(B)**. The images show an untreated A-07 tumor (vehicle; left) and a sunitinib-treated A-07 tumor (sunitinib; right). **C-F**, microvascular density (MVD), hypoxic fraction, necrotic fraction, and IFP in untreated and sunitinib-treated A-07 tumors. Columns, means of 11-15 tumors; bars, SEM.

Sunitinib treatment affected tumor physiology. This is illustrated in Figure 2 which shows representative immunohistochemical preparations stained for microvessels (Figure 2A) and hypoxia (Figure 2B), and graphs illustrating the quantification of microvascular density, hypoxic fraction, necrotic fraction, and tumor IFP in untreated and sunitinib-treated tumors (Figure 2C-F). Sunitinib-treated tumors showed lower microvascular densities (Figure 2C; *P* < 0.0001), higher hypoxic fractions (Figure 2D; *P* = 0.045), and higher necrotic fractions (Figure 2E; *P* = 0.0015) than untreated tumors. Sunitinib-treated tumors did not differ from untreated tumors in IFP (Figure 2F; *P* > 0.05).

**Figure 3:**
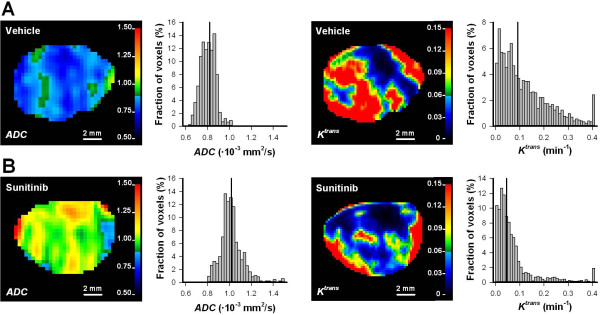
**ADC and *K***^**trans **^**images.** ADC image, the corresponding ADC frequency distribution, *K*^trans^ image, and the corresponding *K*^trans^ frequency distribution of a representative untreated A-07 tumor **(A)** and a representative sunitinib-treated A-07 tumor **(B)**. Color bars show ADC scale in 10^-3^ mm^2^/s or *K*^trans^ scale in min^-1^. Vertical line in the frequency distributions shows median ADC or median *K*^trans^.

**Figure 4:**
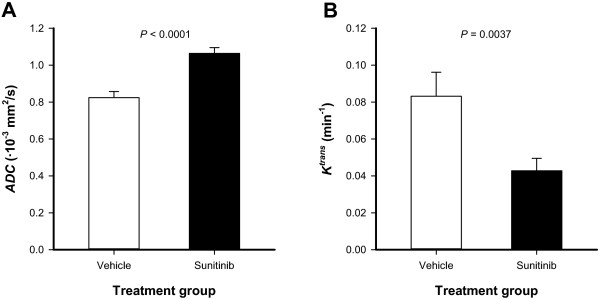
**Sunitinib treatment increased ADC and reduced *K***^**trans **^**values.** ADC **(A)** and *K*^trans^**(B)** in untreated and sunitinib-treated A-07 tumors. Columns, means of 14-15 tumors; bars, SEM.

To investigate whether MRI could detect sunitinb-induced changes in tumor physiology, untreated and sunitinib-treated tumors were subjected to DW-MRI and DCE-MRI. ADC images and ADC frequency distributions were produced from DW-MRI data, and *K*^trans^ images and *K*^trans^ frequency distributions were produced from DCE-MRI series. Figure 3 shows the ADC image, the corresponding ADC frequency distribution, the *K*^trans^ image, and the corresponding *K*^trans^ frequency distribution of a representative untreated tumor (Figure 3A) and a representative sunitinib-treated tumor (Figure 3B). Figure 4 shows average ADC and average *K*^trans^ of 15 untreated and 14 sunitinb-treated tumors, demonstrating that sunitinib-treated tumors showed significantly higher ADC values (Figure 4A; *P* < 0.0001) and significantly lower *K*^trans^ values (Figure 4B; *P* = 0.0037) than untreated tumors.

## Discussion

Sunitinib treatment did not reduce the growth of A-07 tumors, but despite this sunitinib-treated tumors showed altered vasculature and microenvironment and, interestingly, altered ADC and *K*^trans^ values. These observations illustrate that sunitinib treatment affected tumor physiology without affecting tumor size, and that DW-MRI and DCE-MRI were sensitive to these early effects. The observation that this short sunitinib treatment did not affect tumor growth is in line with our previous experience with tumors of the same melanoma line growing in dorsal window chambers [11]. In that study, we observed that 4-days with sunitinib treatment did not affect tumor growth, whereas tumor growth was reduced when the treatment was continued for 8 days.

Treatment-induced reductions in tumor size generally occur late after antiangiogenic treatment [5]. If non-responding patients could be identified shortly after treatment initiation, any ineffective treatment could be stopped to avoid toxicity, and other treatments could be considered. In the current study, a short treatment period was chosen deliberately to investigate whether DW-MRI and DCE-MRI can detect treatment-induced effects occurring before reductions in tumor size. Our study suggests that these MR techniques may be used to identify patients that respond to antiangiogenic treatment before treatment-induced reductions in tumor size can be detected.

Sunitinib-treated tumors showed reduced *K*^trans^ and increased ADC values. The reduction in *K*^trans^ could be attributed to several vascular effects, but sunitinib-induced reduction in microvascular density was probably the dominating effect. We have previously shown that *K*^trans^ reflects vessel density in untreated A-07 tumors [24,28], and in the current study sunitinib-treated tumors showed significantly lower microvascular density than untreated tumors. Sunitinib-induced inhibition of VEGFR-2 may also have reduced vessel permeability, because VEGF-A signaling is known to increase vessel permeability [29]. The reduction in *K*^trans^ may thus also be influenced by reduced vessel permeability. The increase in ADC was probably a result of sunitinib-induced necrosis. Sunitinib-treated tumors showed massive necrosis whereas untreated tumors did not show necrotic regions. Elevated ADC values have been found in necrotic tissue in untreated tumors [12,13], and increases in ADC reflecting treatment-induced necrosis have been reported after chemotherapy, radiation therapy, and treatment with vascular disrupting agents [6].

In the current study, DW-MRI was performed by choosing *b* values of 200-800 s/mm^2^ to avoid confounding effects of blood perfusion, as recommended by Padhani et al. [30]. It is therefore unlikely that the ADC values reported here were significantly influenced by vascular effects. The present study thus strongly suggests that ADC and *K*^trans^ reflected different physiological parameters, illustrating that it may be beneficial to combine DW-MRI and DCE-MRI when evaluating effects of antiangiogenic treatment.

It has been suggested that antiangiogenic agents including sunitininib can normalize tumor vasculature and microenvironment and hence sensitize tumors to conventional therapy [4,31]. Thus antiangiogenic treatment has been shown to enhance blood perfusion, improve oxygenation, and lower IFP in some tumor models [2,3]. In other tumor models, antiangiogenic agents have failed to normalize the vasculature and have induced hypoxia [10,11]. In the current study, sunitinib treatment reduced microvascular density, increased hypoxic fraction, induced necrosis, and did not alter IFP. Consequently, the treatment schedule applied here resulted in changes in the tumor microenvironment that argue against treatment-induced normalization. This observation is in line with our previous experience with A-07 and R-18 human melanoma xenografts growing in dorsal window chambers [11]. In that study, tumors were treated with two different sunitinib doses and the effect was assessed multiple times during the treatment period. The treatments did not improve vascular function at any time point, suggesting that sunitinib cannot normalize tumor vasculature in these melanoma xenografts.

In tumors where antiangiogenic treatment induces hypoxia, neoadjuvant antiangiogenic therapy is expected to reduce the effect of radiation and chemotherapy [7,8]. In contrast, neoadjuvant antiangiogenic therapy has been shown to enhance the effect of radiation or chemotherapy in preclinical tumors where antiangiogenic treatment normalizes the vasculature and the microenvironment [2,3]. The current study suggests that DW-MRI and DCE-MRI can be used to identify tumors where antiangiogenic treatment does not normalize the microenvironment. These tumors respond to antiangiogenic treatment with reduced *K*^trans^ and increased ADC. Interestingly, increased *K*^trans^ and reduced ADC have been reported in tumors where antiangiogenic treatment has normalized the vasculature and the microenvironment [14,32].

Vascular normalization is a transient effect because tumors can switch to other angiogenesis pathways and become resistant to antiangiogenic agents. The duration of improved tumor oxygenation is also expected to be limited because the beneficial effects of vascular normalization may be balanced by severe vascular regression after prolonged exposure to antiangiogenic agents [31]. Winkler et al. demonstrated that VEGFR-2 blockade enhanced the effect of radiation when the tumors were irradiated during the time window when the antiangiogenic agent normalized the vasculature and improved oxygenation [3]. They also showed that VEGFR-2 blockade did not enhance the effect of radiation when tumors were irradiated before or after this time window, suggesting that the timing of combination therapies may be crucial to achieve maximal antitumor effect. Previous studies suggest that DW-MRI and DCE-MRI are sensitive to vascular normalization [14,32], and the current study suggests that these techniques are also sensitive to microenvironmental effects that indicate no normalization. Taken together, these studies suggest that DW-MRI and DCE-MRI may be used to monitor the effect of antiangiogenic treatment to identify a potential normalization window.

## Conclusions

Previous studies have suggested that DW-MRI and DCE-MRI are sensitive to vascular normalization. The current study demonstrates that these techniques also are sensitive to treatment-induced changes in the tumor microenvironment that indicate no normalization, suggesting that these imaging techniques may be used to identify both tumors where antiangiogenic treatment normalizes the microenvironment and tumors where antiangiogenic treatment does not normalize the microenvironment. Furthermore, the current study demonstrates that DW-MRI and DCE-MRI are sensitive to treatment-induced changes in the tumor microenvironment that occur before tumor size is affected, suggesting that these techniques can predict tumor response to antiangiogenic treatment before treatment-induced reductions in tumor size can be detected.

## Competing interests

The authors declare that they have no competing interests.

## Authors’ contributions

JVG, TH, TGS, and EKR conceived and designed the study. JVG, VP, and TH performed the experiments. JVG, VP, TH, and EKR analyzed and interpreted the data. JVG and EKR wrote the manuscript. All authors read and approved the final manuscript.
